# Gastrointestinal diffuse large B-cell lymphoma: Clinical characteristics and prognostic analysis from SEER database

**DOI:** 10.17305/bb.2025.12697

**Published:** 2025-07-08

**Authors:** Fang Du, Lingyun Zhou, Runya Fang, Jiao Chen, Danbo Liu, Hongxian Xiang, Wenyi Lu, Jingsong Wu, Haifei Chen

**Affiliations:** 1 Department of Hematology, Luohu People’s Hospital of Shenzhen, Shenzhen, China; 2 Guangzhou Institute of Cancer Research, The Affiliated Cancer Hospital, Guangzhou Medical University, Guangzhou, China

**Keywords:** Gastrointestinal diffuse large B-cell lymphoma, GI-DLBCL, SEER database, prognostic factors, nomogram, survival analysis.

## Abstract

This study systematically analyzed the clinicopathological characteristics and prognostic factors of gastrointestinal diffuse large B-cell lymphoma (GI-DLBCL) patients using the Surveillance, Epidemiology, and End Results (SEER) database. The Kaplan–Meier method was used for survival analysis, while least absolute shrinkage and selection operator (LASSO) regression analysis was utilized to further filter variables. The Pi for interaction was applied to verify the interactions in the multivariate analysis, and total survival risks were distinguished using hierarchical survival curves. Multivariate Cox regression analysis revealed that hazard ratio (HR) values indicated that age over 60 years (HR ═ 2.85), Ann Arbor stage (stage II: HR ═ 1.22; stage III: HR ═ 1.31; stage IV: HR ═ 1.85), and being widowed (HR ═ 1.40) were independent poor prognostic factors. In contrast, chemotherapy (HR ═ 0.37), radiotherapy (HR ═ 0.84), surgery (HR ═ 0.86), and lymph node resection (HR ═ 0.79) were associated with significant survival benefits. Additionally, an intestinal primary site (HR ═ 0.89), white race (HR ═ 0.78), and other races (HR ═ 0.65) were correlated with better prognosis. The nomogram model constructed from these independent prognostic factors demonstrated excellent predictive performance in both the training and validation cohorts, achieving a concordance index (C-index) of 0.71, significantly outperforming the traditional Ann Arbor staging system, which had a C-index of 0.56. Receiver operating characteristic (ROC) curve analysis indicated high discriminative ability for predicting 3-year, 5-year, and 10-year survival rates, with area under curve (AUC) values of 0.746, 0.756, and 0.756, respectively. Decision curve analysis (DCA) further confirmed the model’s significant clinical net benefit across a wide range of threshold probabilities. The nomogram model developed in this study, based on extensive SEER database data, effectively predicts the prognosis of GI-DLBCL patients and provides a quantitative tool for individualized treatment.

## Introduction

Gastrointestinal diffuse large B-cell lymphoma (GI-DLBCL) is one of the most prevalent forms of extranodal non-Hodgkin lymphoma, comprising approximately 30% to 40% of all extranodal lymphomas [1, 2]. The stomach and intestines serve as primary sites for GI-DLBCL, which presents with highly heterogeneous clinical manifestations and prognoses. Despite standard chemotherapy, some patients remain susceptible to recurrence or progression, resulting in a 5-year overall survival (OS) rate of less than 60% [3, 4]. The prognosis for patients with refractory diffuse large B-cell lymphoma is particularly poor, with a median OS of only 6.3 months and a 2-year survival rate of 20% [5].

Currently, the Ann Arbor staging system is the cornerstone for lymphoma staging; however, its prognostic predictive performance for extranodal lesions, particularly GI-DLBCL, is contentious [6]. The Ann Arbor system may underestimate the influence of the primary site, local treatments (such as surgery or radiotherapy), and patient baseline characteristics (including age and comorbidities) on prognosis [7]. This limitation poses challenges in developing individualized treatment strategies in clinical practice. Previous studies suggest that the Ann Arbor system may be less effective than the TNM staging system in predicting OS for patients with primary gastrointestinal lymphoma [8]. Nonetheless, there remains a lack of effective prognostic prediction models specifically for GI-DLBCL patients.

Nomogram-based approaches and Surveillance, Epidemiology, and End Results (SEER)-based prognostic models for DLBCL have been extensively reported [9–14]. Wang et al. [9] examined prognostic models of GI-DLBCL utilizing the SEER database, Kaplan–Meier survival curves, and nomograms, primarily focusing on OS and median OS. Liu et al. [10] investigated SEER-based prognostic models for primary small intestinal DLBCL, indicating that chemotherapy and surgery are beneficial for survival. A study on primary gastric DLBCL using the SEER database from 1973–2014 revealed that cases from 2001 to 2014 exhibited lower mortality (HR ═ 0.892, *P* ═ 0.001) [11]. Wang et al. [12] developed an SEER-based prognostic model using a dynamic prognostic nomogram to predict the OS of elderly patients with GI-DLBCL. Research on small intestine and colon DLBCL from the SEER database, encompassing 1613 cases, identified age, Ann Arbor stage, marital status (divorced or separated), lack of insurance, and primary colon location as significant prognostic factors [13]. Feng et al. [14] analyzed primary GI-DLBCL using Kaplan–Meier curves and Cox regression analysis, indicating that the five-year OS rates for stomach, small intestine, and colorectum are approximately 50%, with corresponding cancer-specific survival rates around 65%. Multivariate Cox regression identified age, race, marital status, tumor stage, location, and treatment as independent risk factors.

In recent years, studies employing large-sample databases for prognostic modeling have offered new insights into precision medicine in oncology. The SEER database serves as a crucial resource for exploring prognostic factors in rare cancers due to its extensive population coverage, long-term follow-up, and detailed clinical variables [15–17]. This study aims to integrate clinical data of GI-DLBCL patients from the SEER database between 2004 and 2020, analyzing their clinical characteristics and prognostic impact. Based on the analyzed data, a quantifiable nomogram predictive model was constructed to address the existing gap in effective prognostic assessment tools for GI-DLBCL. This model provides evidence-based guidance for identifying high-risk patients and optimizing follow-up strategies, thereby advancing precision medicine in this field.

## Materials and methods

### Data sources and research population

**Figure 1.**
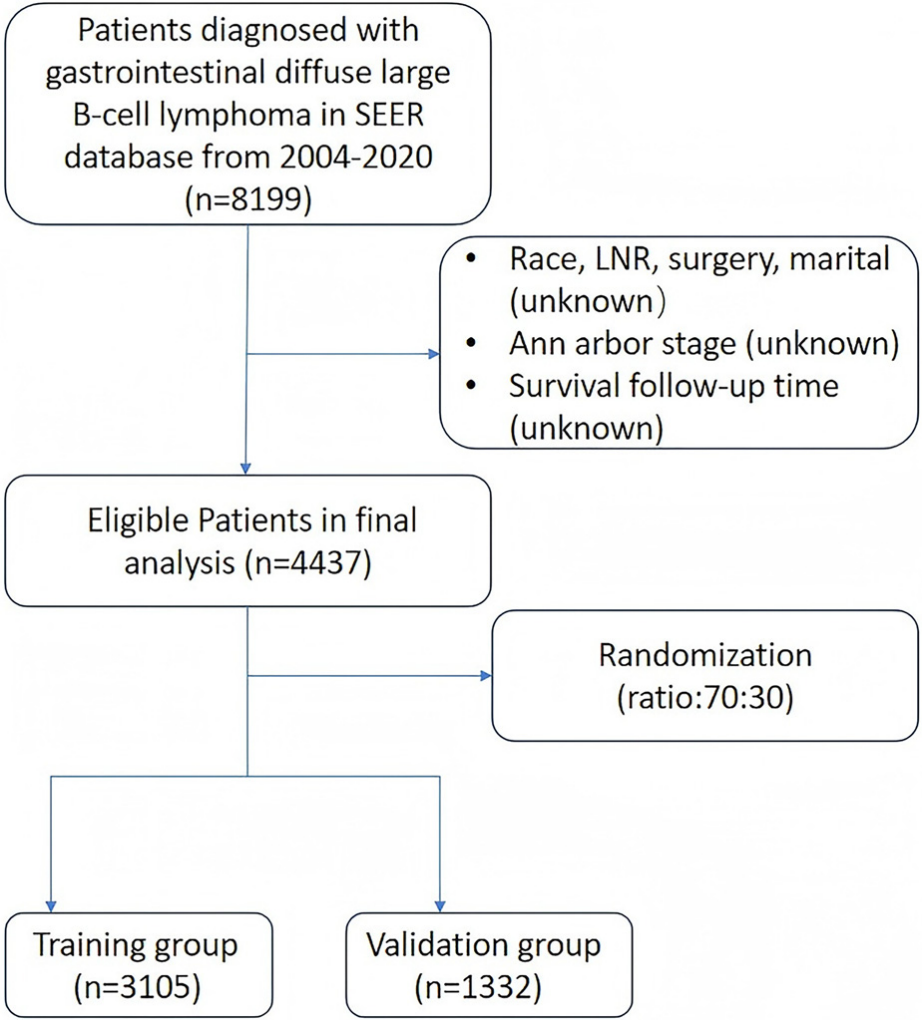
**Flow diagram of patient selection from the SEER database (2004–2020).** Of the 8199 patients diagnosed with GI-DLBCL in the SEER database, 3762 were excluded due to missing data on race, surgery, marital status, Ann Arbor stage, or survival follow-up. A total of 4437 eligible patients were included and randomized into training (*n* ═ 3105) and validation (*n* ═ 1332) cohorts using a 70:30 ratio. GI-DLBCL: Gastrointestinal diffuse large B-cell lymphoma; SEER: Surveillance, Epidemiology, and End Results.

The data for this study were obtained from the SEER database established by the US National Cancer Institute. Patients diagnosed with GI-DLBCL between 2004 and 2020 were extracted using SEER*Stat software (version 8.4.1). Inclusion criteria comprised: (1) confirmed pathological diagnosis of DLBCL; (2) primary site located in the stomach or intestine; (3) availability of complete clinical information and follow-up data. Exclusion criteria included: (1) missing data on race, lymph node dissection status, surgical history, and marital status; (2) unknown Ann Arbor stage; (3) lack of survival follow-up time. The screening process is illustrated in Figure 1.

### Data collection

Demographic and clinicopathological data of patients were collected, including age, sex, race (white, black, other), marital status, primary site (stomach or intestine), Ann Arbor stage, treatment modalities (chemotherapy, radiotherapy, surgery), lymph node resection (LNR), and income level (≤$75,000 or >$75,000). The predefined research endpoint is OS, defined as the duration from diagnosis to death or the last follow-up. Survival status and survival time data were obtained from the SEER database.

### Statistical analysis

Statistical analysis was conducted using R language (version 4.2.2). Categorical data were expressed as frequencies and percentages, and the χ^2^ test was employed for group comparisons. The Kaplan–Meier method was utilized to generate survival curves, while the Log-rank test assessed survival differences across various variables. Least absolute shrinkage and selection operator (LASSO) regression analysis was performed to refine the selection of variables, and interaction effects were examined using Pi for interaction. Total survival risks were differentiated through hierarchical survival curves. Additionally, a Cox proportional hazards regression model was applied for both univariate and multivariate analyses to identify independent risk factors influencing OS. Based on the findings from the multivariate Cox regression, a nomogram was developed to predict individual survival probabilities at 3, 5, and 10 years. The model’s discriminative ability was evaluated using the concordance index (C-index) and the AUC of the ROC curve. The consistency between the predicted survival probabilities and the actual observed survival rates was further validated through calibration curves. Furthermore, decision curve analysis (DCA) was employed to quantify the clinical net benefit of the model across varying risk thresholds, thereby assessing its clinical applicability. Statistical significance was set at *P* < 0.05.

## Results

### Basic clinical features

A total of 4437 patients diagnosed with GI-DLBCL in the SEER database from 2004 to 2020 were randomly assigned to a training group (3105 cases) and a verification group (1332 cases) in a 7:3 ratio. Among these patients, 59.91% were male and 40.09% were female, with 71.87% being over 60 years of age. The majority of patients identified as white (80.32%), while other races and Black patients comprised 13.16% and 6.51%, respectively. Regarding the primary sites of the disease, the stomach and intestine accounted for 50.98% and 49.02%, respectively. The distribution of Ann Arbor stages was primarily I (43.79%) and II (26.77%). Of the patients, 69.53% received chemotherapy, 36.74% underwent surgery, and 17.80% had lymphadenectomy. Additionally, 40.43% of patients reported an income exceeding $75,000. The training and verification groups were evenly distributed across these variables, with no statistically significant differences (all *P* ≥ 0.05), as illustrated in Table 1.

**Table 1:** Comparison of basic characteristics between the train group and the validation group

**Variables**	**Total (*n* ═ 4437)**	**Validation (*n* ═ 1332)**	**Train (*n* ═ 3105)**	**Statistic**	***P***
Sex, *n* (%)				χ^2^ ═ 0.00	0.997
Female	1779 (40.09)	534 (40.09)	1245 (40.10)		
Male	2658 (59.91)	798 (59.91)	1860 (59.90)		
Race, *n* (%)				χ^2^ ═ 0.90	0.639
Black	289 (6.51)	87 (6.53)	202 (6.51)		
Others	584 (13.16)	185 (13.89)	399 (12.85)		
White	3564 (80.32)	1060 (79.58)	2504 (80.64)		
Radiotherapy, *n* (%)				χ^2^ ═ 0.00	0.968
None/Unknown	3922 (88.39)	1177 (88.36)	2745 (88.41)		
Yes	515 (11.61)	155 (11.64)	360 (11.59)		
Chemotherapy, *n* (%)				χ^2^ ═ 0.84	0.36
No/Unknown	1352 (30.47)	393 (29.50)	959 (30.89)		
Yes	3085 (69.53)	939 (70.50)	2146 (69.11)		
Marital, *n* (%)				χ^2^ ═ 3.97	0.41
Divorced	310 (6.99)	107 (8.03)	203 (6.54)		
Married	2536 (57.16)	747 (56.08)	1789 (57.62)		
Separated	35 (0.79)	10 (0.75)	25 (0.81)		
Single	762 (17.17)	236 (17.72)	526 (16.94)		
Widowed	794 (17.89)	232 (17.42)	562 (18.10)		
Ann Arbor stage, *n* (%)				χ^2^ ═ 0.18	0.98
Stage I	1943 (43.79)	577 (43.32)	1366 (43.99)		
Stage II	1188 (26.77)	360 (27.03)	828 (26.67)		
Stage III	315 (7.10)	96 (7.21)	219 (7.05)		
Stage IV	991 (22.33)	299 (22.45)	692 (22.29)		
Age, *n* (%)				χ^2^ ═ 1.25	0.264
≤60	1248 (28.13)	390 (29.28)	858 (27.63)		
>60	3189 (71.87)	942 (70.72)	2247 (72.37)		
Primary site, *n* (%)				χ^2^ ═ 0.35	0.553
Stomach	2262 (50.98)	670 (50.30)	1592 (51.27)		
Intestine	2175 (49.02)	662 (49.70)	1513 (48.73)		
Surgery, *n* (%)				χ^2^ ═ 0.10	0.751
No	2807 (63.26)	838 (62.91)	1969 (63.41)		
Yes	1630 (36.74)	494 (37.09)	1136 (36.59)		
LNR, *n* (%)				χ^2^ ═ 3.50	0.061
No	3647 (82.20)	1073 (80.56)	2574 (82.90)		
Yes	790 (17.80)	259 (19.44)	531 (17.10)		
Income, *n* (%)				χ^2^ ═ 1.37	0.241
≤$75,000	2643 (59.57)	811 (60.89)	1832 (59.00)		
>$75,000	1794 (40.43)	521 (39.11)	1273 (41.00)		

LNR: Lymph node ratio; χ^2^: Chi-square.

### Influencing factors of prognosis in patients with GI-DLBCL

Univariate Cox regression analysis indicated that age, race, radiotherapy, chemotherapy, operation, LNR, primary site, Ann Arbor stage, and marital status significantly influenced the prognosis of patients with GI-DLBCL (*P* < 0.05). Multivariate analysis identified the following independent prognostic factors: age greater than 60 years (HR ═ 2.85, 95% CI: 2.49–3.26, *P* < 0.001) and advanced Ann Arbor stages (HR ═ 1.31 for stage III, HR ═ 1.85 for stage IV, *P* < 0.001), both associated with poor prognosis. Specifically, patients older than 60 years had a mortality risk 2.85 times higher than younger patients (HR ═ 2.85, 95% CI: 2.49–3.26, *P* < 0.001).

After adjusting for age, being widowed remained significantly correlated with increased mortality risk (HR ═ 1.40, 95% CI: 1.14–1.72, *P* ═ 0.001). Interaction testing yielded a *P* value of 0.099 for the interaction between marital status and age, indicating that the interaction effect was not statistically significant. The true hazard ratio (HR) is likely to fall within the confidence interval of 2.49–3.26, and since this interval does not include 1 and *P* < 0.001, it underscores the highly statistically significant impact of age on prognosis, suggesting a marked deviation from “no impact” (where HR ═ 1).

Patients who underwent chemotherapy (HR ═ 0.37, *P* < 0.001), radiotherapy (HR ═ 0.84, *P* ═ 0.023), surgical procedures (HR ═ 0.86, *P* ═ 0.032), had a lower LNR (HR ═ 0.79, *P* ═ 0.002), or had a primary tumor site in the intestine (HR ═ 0.89, *P* ═ 0.037) exhibited better prognoses. Furthermore, compared to Black patients, White patients and those of “other” races showed improved prognoses (HR ═ 0.65 and HR ═ 0.78, respectively, *P* < 0.01), as detailed in Table 2. For race, gender, marital status, Ann Arbor stages II and III, and income levels exceeding $75,000, the HR range included 1 and *P* ≥ 0.05, suggesting that survival is influenced by multiple factors (including age and treatment modalities), short follow-up duration, and limited sample sizes (e.g., among separated individuals, Black patients, and those in Ann Arbor stage III).

**Table 2:** Univariate and multivariate Cox regression analysis of prognosis of GI-DLBCL patients in the training cohort

**Variables**	**Univariate analysis**		**Multivariate analysis**	
	HR (95% CI)^*^	***P***	**HR (95% CI)**	***P***
*Sex*				
Female	1.00 (Reference)			
Male	0.98 (0.90 ∼ 1.08)	0.733		
*Race*				
Black	1.00 (Reference)		1.00 (Reference)	
Others	0.77 (0.62 ∼ 0.96)	0.018	0.65 (0.52 ∼ 0.81)	<.001
White	0.93 (0.78 ∼ 1.12)	0.457	0.78 (0.65 ∼ 0.94)	0.007
*Radiotherapy*				
None/Unknown	1.00 (Reference)		1.00 (Reference)	
Yes	0.75 (0.65 ∼ 0.87)	<.001	0.84 (0.72 ∼ 0.98)	0.023
Chemotherapy				
No/Unknown	1.00 (Reference)		1.00 (Reference)	
Yes	0.38 (0.35 ∼ 0.42)	<.001	0.37 (0.33 ∼ 0.41)	<.001
*Marital*				
Divorced	1.00 (Reference)		1.00 (Reference)	
Married (2536)	0.95 (0.78 ∼ 1.14)	0.56	0.99 (0.82 ∼ 1.20)	0.923
Separated 35	0.51 (0.26 ∼ 1.01)	0.052	1.04 (0.53 ∼ 2.05)	0.912
Single 762	0.85 (0.69 ∼ 1.06)	0.152	1.22 (0.98 ∼ 1.51)	0.077
Widowed	1.83 (1.49 ∼ 2.24)	<.001	1.40 (1.14 ∼ 1.72)	0.001
*Ann Arbor stage*				
Stage I	1.00 (Reference)		1.00 (Reference)	
Stage II	0.94 (0.84 ∼ 1.05)	0.267	1.22 (1.08 ∼ 1.37)	<.001
Stage III	1.01 (0.84 ∼ 1.22)	0.905	1.31 (1.08 ∼ 1.58)	0.005
Stage IV	1.52 (1.36 ∼ 1.70)	<.001	1.85 (1.65 ∼ 2.08)	<.001
*Age*				
≤60	1.00 (Reference)		1.00 (Reference)	
>60	3.13 (2.75 ∼ 3.55)	<.001	2.85 (2.49 ∼ 3.26)	<.001
*Primary site*				
Stomach	1.00 (Reference)		1.00 (Reference)	
Intestine	0.85 (0.78 ∼ 0.93)	<.001	0.89 (0.79 ∼ 0.99)	0.037
*Surgery*				
No	1.00 (Reference)		1.00 (Reference)	
Yes	0.89 (0.81 ∼ 0.98)	0.015	0.86 (0.75 ∼ 0.99)	0.032
*LNR*				
No	1.00 (Reference)		1.00 (Reference)	
Yes	0.84 (0.74 ∼ 0.95)	0.004	0.79 (0.68 ∼ 0.92)	0.002
*Income*				
≤75,000	1.00 (Reference)			
>75,000	0.91 (0.83 ∼ 1.00)	0.056		

^*^HR (95% CI): Hazard Ratio (HR), 95% (Confidence Interval, CI), HR is within the CI range with 95% probability. *Note:* HR, HR > 1 is a risk factor (such as age >60 years, stage IV, widowed), HR < 1 is a protective factor (such as chemotherapy and primary site at intestine), HR ═ 1 is no effect (such as black ethnicity in multiple factors). 95% CI excluding 1 indicate results are statistically significant, and including 1 indicate results are not statistically significant. The HR ═ 1 (reference) are defined as reference values for comparison with independent risk factors. Probability value (*P*) <0.05 suggest statistically significant, *P* ≥ 0.05 suggest not statistically significant. GI-DLBCL: Gastrointestinal diffuse large B-cell lymphoma; LNR: Lymph node ratio.

Although high income levels (>$75,000, HR ═ 0.91) were identified as a potential protective factor in univariate analysis, the *P* value of 0.056 indicated that this finding was not statistically significant; therefore, it was not included in multivariate analysis.

When constructing a Cox proportional hazards regression model, variable selection must balance statistical significance (*P* < 0.05) with clinical and biological relevance to avoid false associations and overfitting that may arise from simple stepwise regression. LASSO regression employs L1 regularization (the LASSO penalty term) to compress variable coefficients (β). As the penalty intensity (λ) increases, some coefficients are reduced to zero, facilitating variable selection.

**Figure 2.**
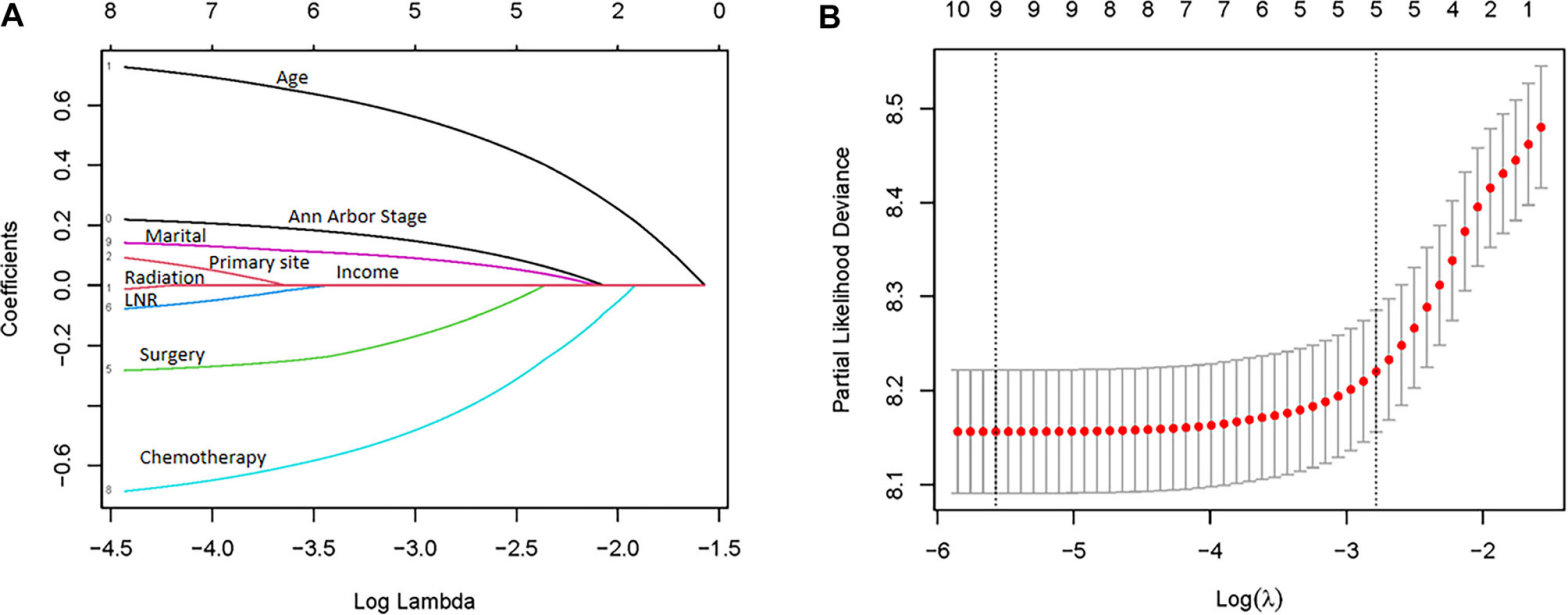
**LASSO regression analysis of different variables.** (A) The coefficient path plot of variable coefficients as a function of Log *λ* (where *λ* is the penalty intensity), reflecting the contraction process of variable coefficients β (vertical axis) under different penalty intensities. Positive β indicate a positive correlation between variables and risk factors. (B) Partial likelihood deviance under cross validation, which is used to select the optimal value of λ.

As illustrated in Figure 2, LASSO regression identifies Age (β ═ 0.774, positively correlated with risk) and Chemotherapy (β ═ –0.733, negatively correlated with risk) as the two most significant variables, aligning with the findings from the multivariate Cox regression analysis, which noted age >60 years (HR ═ 2.85) and chemotherapy (HR ═ 0.37) as significant factors. Additionally, Ann Arbor Stage (β ═ 0.235), Primary Site (β ═ 0.146), and Marital Status (β ═ 0.157) positively influence risk factors, while Surgery (β ═ –0.302) and LNR (β ═ –0.114) may enhance prognosis.

The coefficients for Radiotherapy (β ═ –0.02) and Income (β ═ –0.04) are close to zero, and the coefficients for Sex and Race are exactly zero, indicating minimal impact on risk factors. In comparison to the Cox regression results presented in Table 2, the effects of Ann Arbor Stage, Marital Status, Race, and Radiotherapy in the LASSO regression analysis exhibit inconsistencies with respect to risk factors, as LASSO regression does not account for variable stratification. For instance, Marital Status (β ═ 0.157) is positively associated with risk in LASSO regression; however, Cox regression indicates that different marital statuses have varying impacts on risk, including Married (*P* ═ 0.56), Separated (*P* ═ 0.052), Single (*P* ═ 0.152), and Widowed (*P* < 0.001), with only the Widowed category demonstrating a negative effect on survival rates.

**Figure 3.**
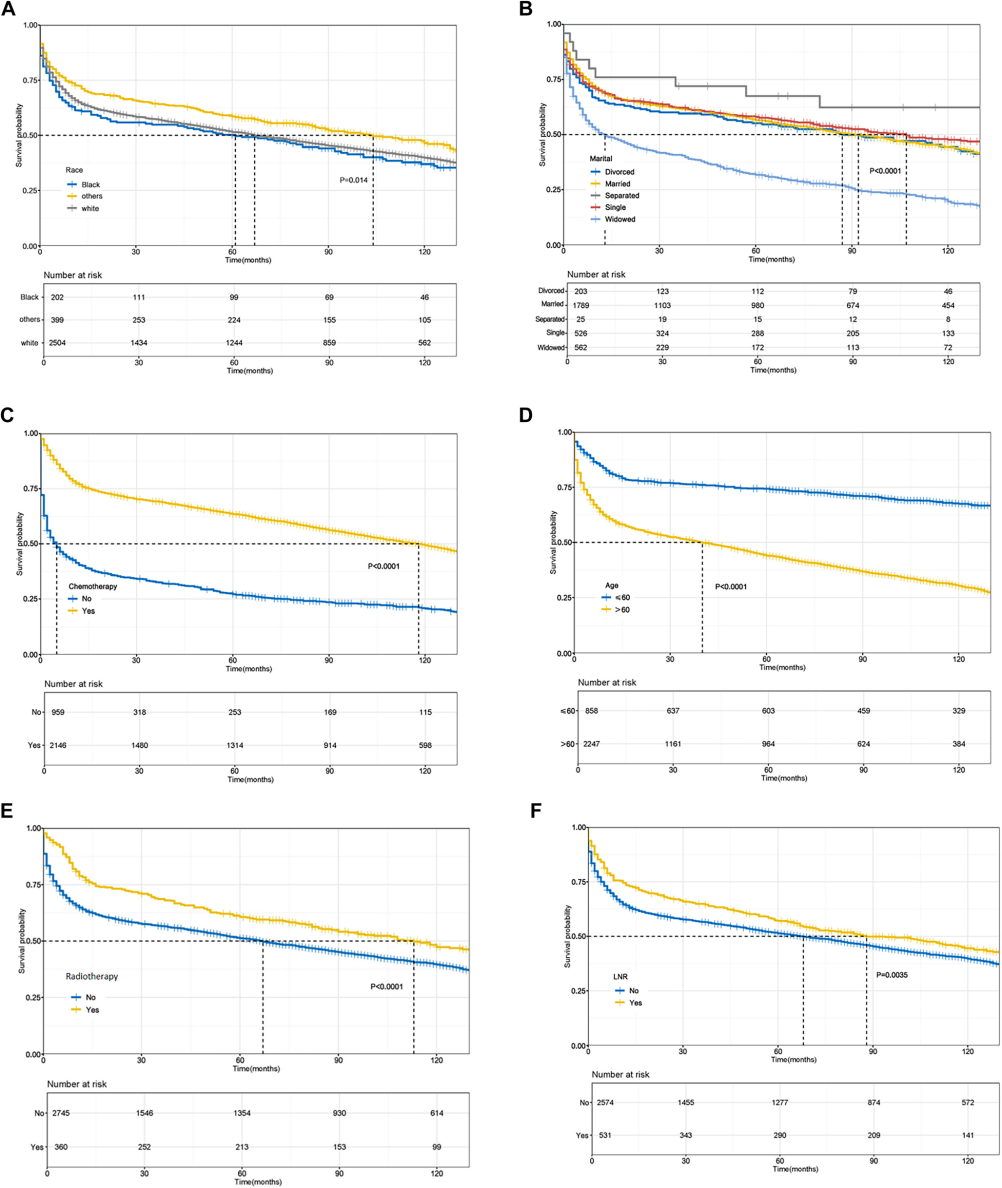
**Kaplan–Meier survival curves for six independent prognostic factors in patients with GI-DLBCL.** Vertical dashed lines represent median follow-up time. (A) Race; (B) Marital status; (C) Chemotherapy; (D) Age; (E) Radiotherapy; (F) LNR. GI-DLBCL: Gastrointestinal diffuse large B-cell lymphoma; LNR: Lymph node resection.

**Figure 4.**
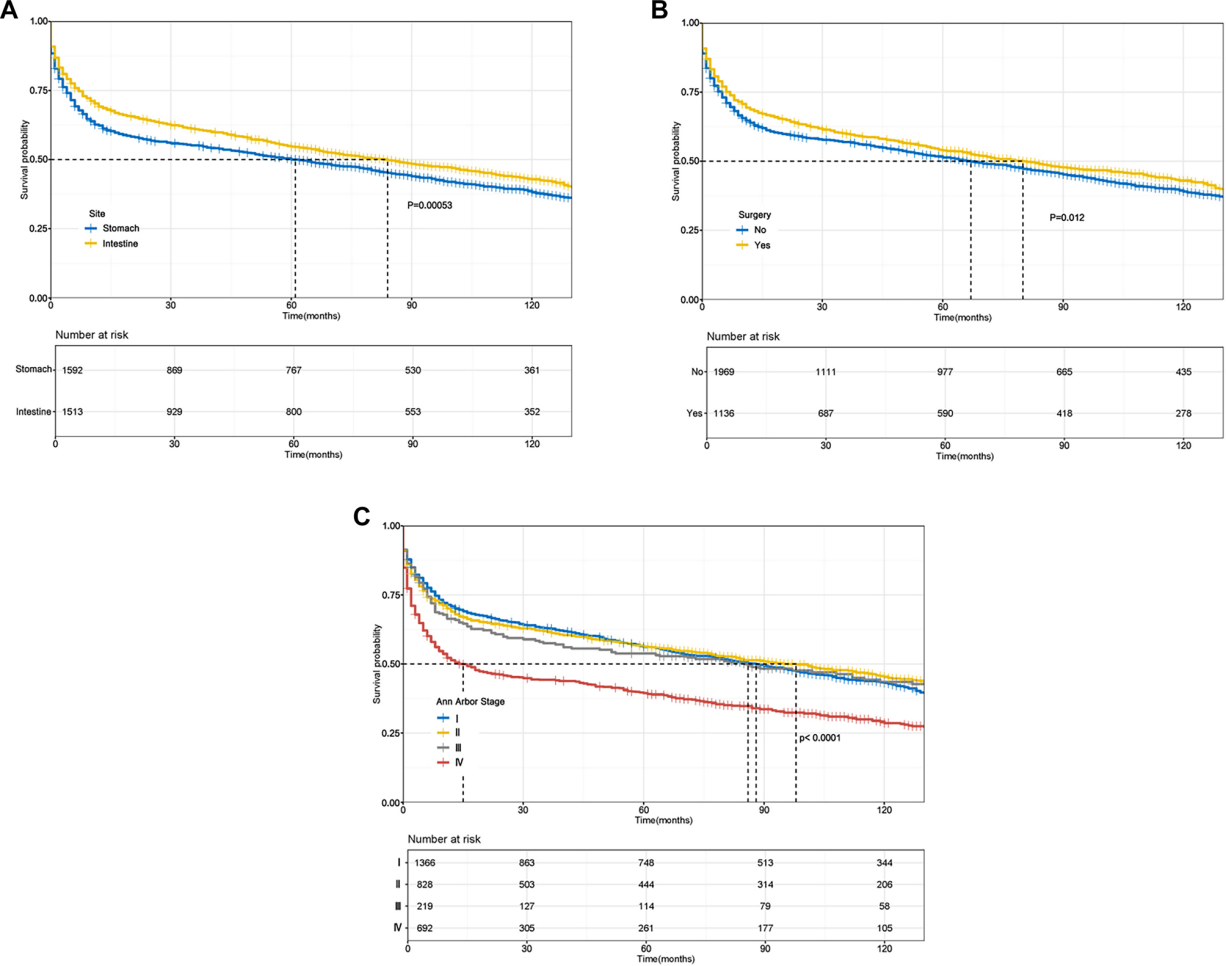
**Kaplan–Meier survival curves for three independent prognostic factors in patients with GI-DLBCL.** Vertical dashed lines represent median follow-up time. (A) Primary site; (B) Surgery; (C) Ann Arbor stage. GI-DLBCL: Gastrointestinal diffuse large B-cell lymphoma.

The survival curves for the nine independent prognostic factors are depicted in Figures 3 and 4. Notably, the survival rates for elderly patients, those with late Ann Arbor stage, and widowed individuals exhibited a significant downward trend. A multivariate survival analysis was conducted using the Kaplan–Meier method, incorporating nine independent prognostic factors (*P* < 0.05): race, marital status, chemotherapy, age, primary site, surgery, and Ann Arbor stage. The observed survival differences across various groups were statistically significant, suggesting that these factors can independently predict patient prognosis.

Age, income, marital status, and race may be associated with competitive risks, indicating that univariate Cox models may not adequately account for the effects of these variables. Fine-gray subdistribution and multivariate Cox model risk analyses reveal that age and treatment method, race/income and treatment accessibility, LNR and Ann Arbor stage, and marital status and treatment compliance represent competitive risks for survival. For instance, the proportion of widowed patients over 60 is elevated; elderly patients (≥60 years) often exhibit poor tolerance to radiotherapy and chemotherapy. Additionally, low-income or ethnic minority patients may interrupt treatment due to economic constraints and face a higher burden of complications. Conversely, married patients generally demonstrate higher treatment compliance, while late Ann Arbor stage lesions are more susceptible to systemic metastasis. The interaction *P* value assesses whether a variable significantly modifies the effect of another variable, as determined by statistical differences in HRs across subgroups. An interaction *P* value < 0.05 indicates a statistically significant interaction, while a value ≥ 0.05 suggests no significant interaction. In the interaction analysis of radiotherapy with other variables, only the primary site demonstrated a statistically significant interaction (interaction *P* value < 0.05). In contrast, the interaction analysis between chemotherapy and other variables revealed significant interactions with both Ann Arbor stage and primary site (interaction *P* value < 0.05). Regarding surgery, significant interactions were observed with both primary site and LNR (interaction *P* value < 0.05). Similarly, the interaction analysis between LNR and other variables showed significant interactions with primary site, Ann Arbor stage, and surgery, all exhibiting interaction *P* values below 0.05. In the interaction analysis between age and other variables, only sex and Ann Arbor stage showed significant interactions, with interaction *P* values < 0.05.

**Figure 5.**
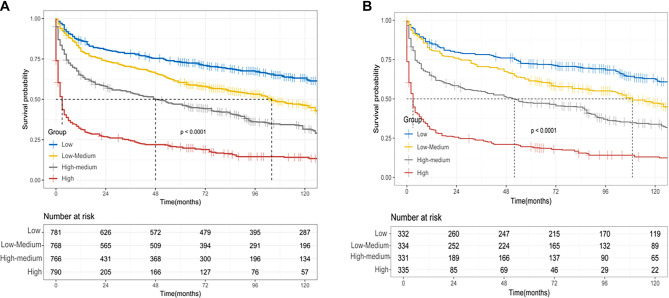
**All the multivariate factors related Kaplan–Meier hierarchical survival curves.** (A) Training group; (B) Validation group.

The multivariate factor-related survival curve analysis (Figure 5) demonstrates that the survival curve trends for the training and validation groups are highly consistent, indicating that the “risk stratification” effect is replicable across different groups. The survival probability displays a gradient decrease over time across four groups (low, low median, high median, high) in both Figures 5A and 5B. The intergroup differences are statistically significant, with *P* < 0.0001. Stratification based on a comprehensive multivariate analysis effectively distinguishes the survival risk among patients. The low-risk group exhibits a high survival probability and slow event accumulation, while the high-risk group demonstrates a low survival probability and rapid event accumulation.

### Establishment and verification of nomogram

**Figure 6.**
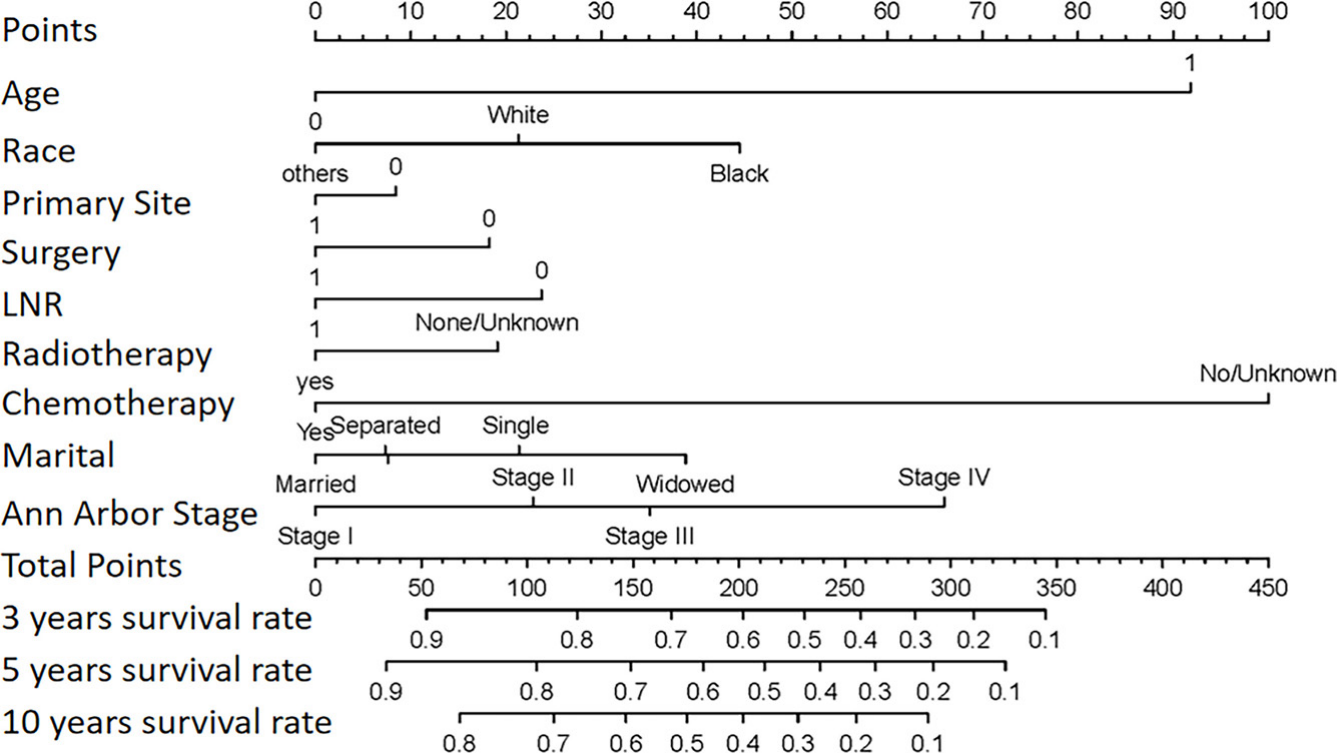
**Nomogram for predicting overall survival in patients with GI-DLBCL.** The nomogram was constructed based on independent prognostic factors identified through multivariate Cox regression analysis, including age, race, primary tumor site, surgery, LNR, radiotherapy, chemotherapy, marital status, and Ann Arbor stage. Each variable is assigned a point value, and the total points correspond to predicted 3-, 5-, and 10-year overall survival probabilities. Higher total points indicate a poorer prognosis. The model enables individualized survival prediction for patients with GI-DLBCL. GI-DLBCL: Gastrointestinal diffuse large B-cell lymphoma; LNR: Lymph node resection.

According to independent prognostic factors identified through multivariate Cox regression analysis—including age, race, primary site, surgery, LNR, radiotherapy, chemotherapy, marital status, and Ann Arbor stage—a nomogram model was developed to predict the 3-year, 5-year, and 10-year OS rates of patients with gastrointestinal as illustrated in Figure 6. Each variable is assigned a corresponding score based on its regression coefficient, allowing for the prediction of individual survival probabilities by summing the scores of patients. In the nomogram, scores ranging from 0–100 represent the univariate risk of death, while total scores from 0–450 reflect the cumulative multivariate risks. The arrangement of axes allocates higher scores to higher-risk categories. For instance, white and “other” racial groups exhibit better prognoses than black individuals, resulting in a higher risk of death for blacks, which is reflected in their elevated scores.

In evaluating the model’s performance, the nomogram achieved a C-index of 0.71 (95% CI: 0.70–0.73) in the training group and 0.71 (95% CI: 0.69–0.73) in the validation group, indicating excellent discrimination capability. The difference between the nomogram and Ann Arbor stage is defined as ΔC, which was calculated to be 0.15. Compared to the traditional Ann Arbor stage, the predictive performance of the nomogram exhibits a relative improvement of approximately 26.8%. In contrast, the traditional Ann Arbor staging system yielded a C-index of only 0.56 (95% CI: 0.54–0.57), with a statistically significant difference (*P* < 0.001) (Table 3).

**Table 3:** C-index of the nomogram model and Ann Arbor staging system

**Classification**	**Training group**		**Validation group**	
	**C-index (95% CI)**	***P* value**	**C-index (95% CI)**	***P* value**
Nomogram	0.71 (0.70–0.73)	1	0.71 (0.69–0.73)	1
Ann Arbor stage	0.56 (0.54–0.57)	<0.001	0.56 (0.54–0.57)	<0.001
ΔC-index (Nomogram - Ann Arbor)	0.15	/	0.15	/

C-index: Concordance index.

**Figure 7.**
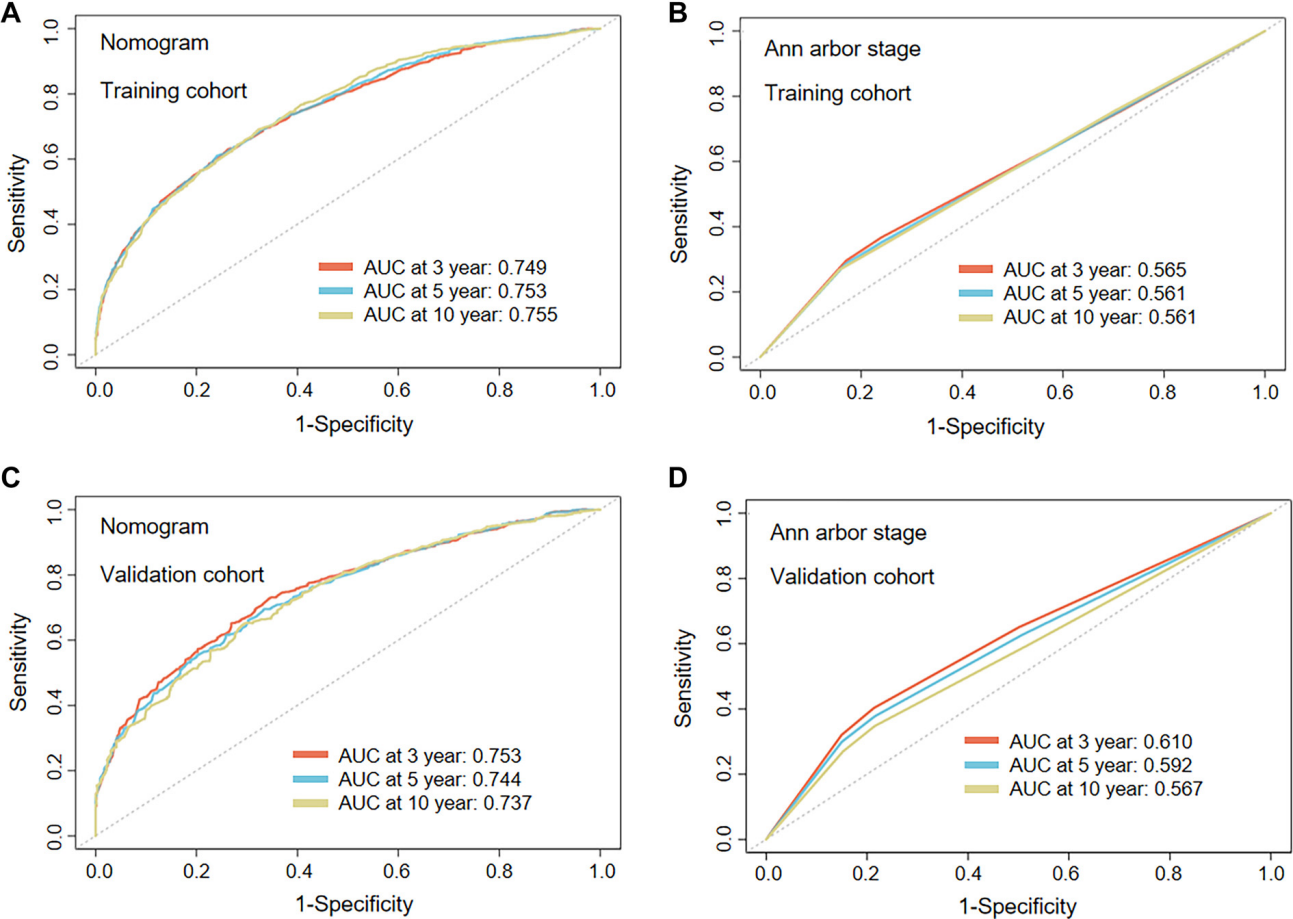
**ROC curve analysis of the prognostic predictive performance of the nomogram model versus the Ann Arbor staging system in patients with GI-DLBCL.** (A and B) ROC curves and AUC values for predicting 3-, 5-, and 10-year overall survival in GI-DLBCL patients from the training cohort, where (A) represents the nomogram model and (B) represents the Ann Arbor staging system; (C and D) ROC curves and AUC values for predicting 3-, 5-, and 10-year overall survival in GI-DLBCL patients from the internal validation cohort, where (C) represents the nomogram model and (D) represents the Ann Arbor staging system. ROC: Receiver operating characteristic; GI-DLBCL: Gastrointestinal diffuse large B-cell lymphoma; AUC: Area under curve.

The ROC curve further demonstrates that the nomogram developed in this study possesses superior discriminatory power in predicting OS compared to the traditional Ann Arbor stage model. Figure 7 presents a comparison of the predictive performance between the nomogram and the Ann Arbor staging system in both the training cohort (A, C) and the internal validation cohort (B, D), confirming that the nomogram exhibits significant advantages at various time points (3, 5, and 10 years). Notably, in long-term prognosis assessment (10-year survival), the nomogram maintains stable performance with AUC > 0.73, while the predictive performance of traditional staging systems declines significantly over time (10-year AUC is only 0.56). The 95% CIs for all AUC values are detailed in Table 4.

**Table 4:** AUC of the nomogram model and Ann Arbor staging system

**Classification**	**Training group**	**Validation group**
	**AUC (95% CI)**	**AUC (95% CI)**
Nomogram (3 years)	0.749 (0.71–0.81)	0.753 (0.72–0.82)
Nomogram (5 years)	0.753 (0.72–0.84)	0.744 (0.68–0.80)
Nomogram (10 years)	0.755 (0.73–0.88)	0.737 (0.65–0.79)
Ann Arbor stage (3 years)	0.565 (0.52–0.63)	0.610 (0.58–0.66)
Ann Arbor stage (5 years)	0.561 (0.50–0.62)	0.592 (0.54–0.65)
Ann Arbor stage (10 years)	0.561 (0.49–0.62)	0.567 (0.52–0.64)

**Figure 8.**
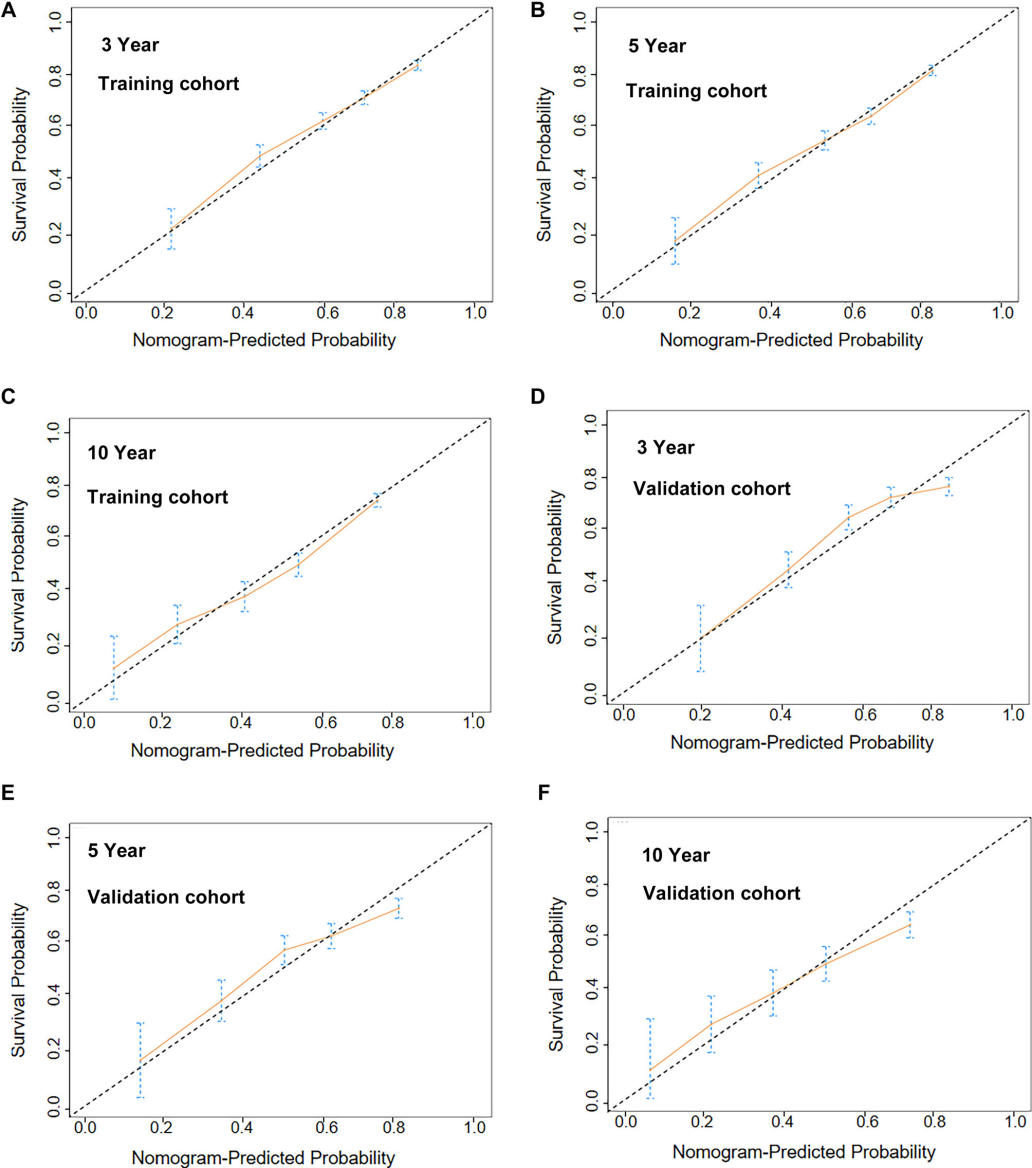
**Calibration curve analysis of the nomogram model for predicting overall survival in patients with GI-DLBCL.** (A–C) Calibration curves for predicting 3-, 5-, and 10-year overall survival in GI-DLBCL patients from the training cohort; (D–F) Calibration curves for predicting 3-, 5-, and 10-year overall survival in GI-DLBCL patients from the internal validation cohort. GI-DLBCL: Gastrointestinal diffuse large B-cell lymphoma.

Figure 8 illustrates the calibration curve analysis of the nomogram model. The dashed line represents the ideal prediction scenario, with closer scatter distributions indicating greater consistency between the model’s predicted probabilities and actual survival rates. In the training cohort (A–C), the predicted lines for 3, 5, and 10 years closely follow the dashed line, indicating excellent fit within the training data. In the validation cohort (D–F), while the overall trend of the predicted line aligns with the dashed line, deviations are larger than those observed in the training cohort. The calibration curve confirms that the OS probabilities predicted by the nomogram model closely match actual observed values, demonstrating strong calibration.

**Figure 9.**
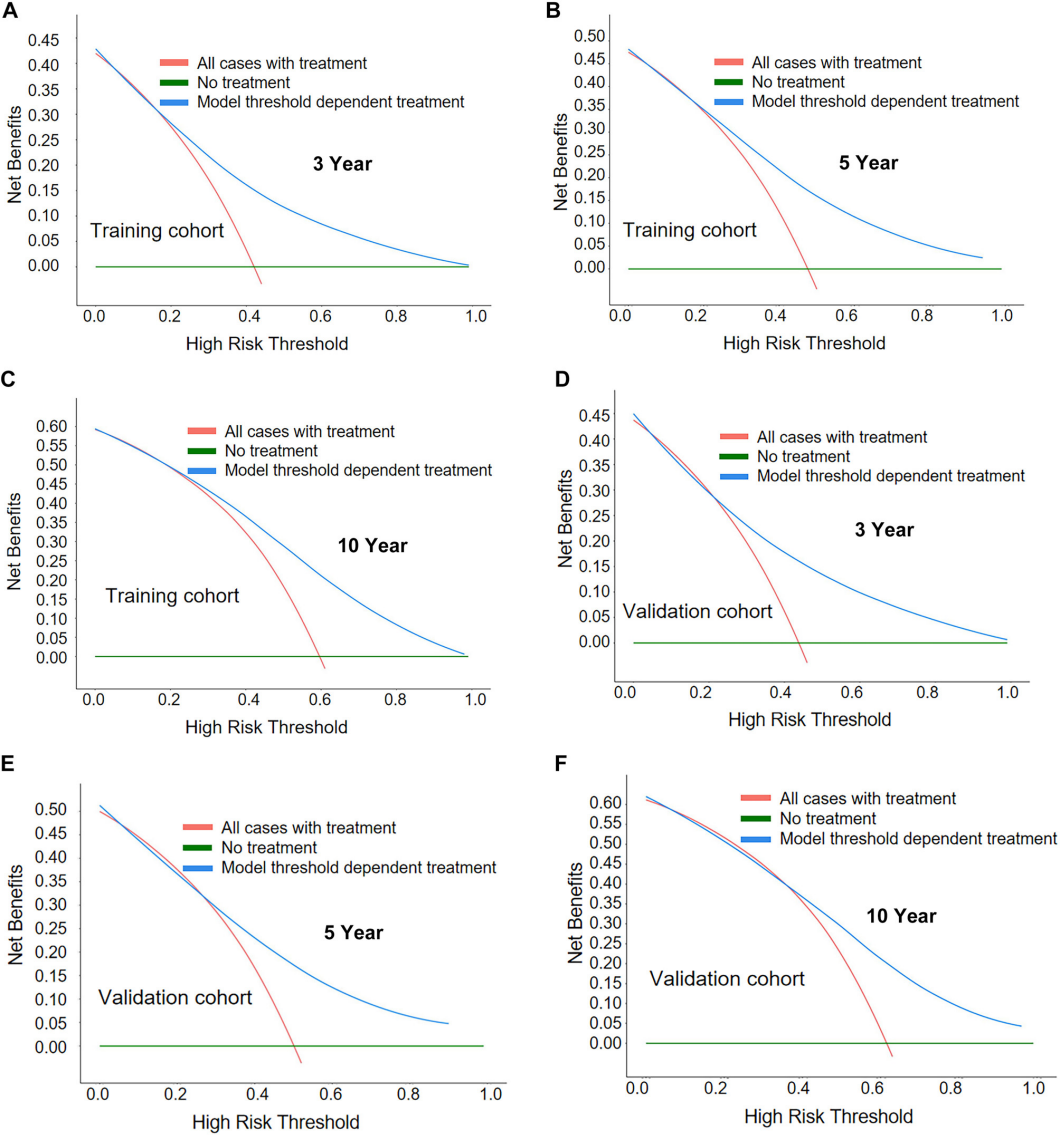
**DCA of the nomogram model for clinical utility in predicting survival of patients with GI-DLBCL.** (A–C) DCA for the 3-, 5-, and 10-year survival prediction models in GI-DLBCL patients from the training cohort; (D–F) DCA for the 3-, 5-, and 10-year survival prediction models in GI-DLBCL patients from the internal validation cohort. DCA: Decision curve analysis; GI-DLBCL: Gastrointestinal diffuse large B-cell lymphoma.

Figure 9 presents the DCA of the nomogram model, where the “all” reference strategy indicates treatment in all cases, while the “none” reference strategy indicates no treatment. The clinical applicability of the GI-DLBCL patient survival prediction nomogram is validated by comparing the net benefits of three decision strategies across different risk thresholds in the training and internal validation cohorts. The DCA results indicate a positive net benefit for this model, suggesting significant clinical application potential for the nomogram constructed in this study.

## Discussion

Based on extensive data from the SEER database, this study systematically analyzes the clinical characteristics and prognostic factors of patients with GI-DLBCL, and constructs a nomogram model that integrates variables such as age, stage, treatment method, and social demographics. The findings indicate that age over 60 years, late Ann Arbor stage, and widowed status are independent adverse prognostic factors. Conversely, receiving systematic treatment (chemotherapy, radiotherapy, surgery, and lymphadenectomy) and being diagnosed with primary intestinal tract involvement are significantly associated with improved survival outcomes.

This study highlights that advanced age (greater than 60 years) significantly negatively impacts prognosis (HR ═ 2.85). Gao et al. also identified advanced age as a critical prognostic factor, likely [18]. This may be related to the decline in immune function, increased comorbidities, and reduced treatment tolerance in older patients. Furthermore, elderly patients with DLBCL may experience diminished therapeutic efficacy due to inadequate dose adjustments or interruptions in treatment. Wang et al. [12] developed a dynamic prognostic nomogram model specifically for elderly GI-DLBCL patients, enhancing prognostic accuracy.

The Ann Arbor staging system, a traditional prognostic indicator, retains independent predictive value in this analysis. However, the mortality risk for stage IV patients (HR ═ 1.85) is significantly higher than previously reported [19], suggesting that the extranodal aggressiveness of GI-DLBCL may be underestimated. Further stratification using imaging or molecular markers is warranted [7]. Notably, widowed status (HR ═ 1.40) is identified for the first time as an independent risk factor, potentially linked to insufficient social support, psychological stress, or delays in medical care [20, 21]. Further studies are needed to validate its biological or social mechanisms. Additionally, both cross-sectional and longitudinal studies indicate a significant association between widowhood and cognitive function decline, such as impaired executive function and reduced decision-making ability [22]. This may further weaken patients’ adaptability to complex treatment regimens, increase disease management burdens, and affect the outcome of the disease.

In terms of therapeutic interventions, chemotherapy demonstrates the most significant survival benefit (HR ═ 0.37), aligning with the established efficacy of the R-CHOP regimen in DLBCL [23]. However, the HR value of radiotherapy and surgery is close to 1 (0.84 and 0.86), suggesting that its effect may be limited to a specific subgroup (such as patients with locally advanced stage or chemotherapy intolerance) [24]. The prognostic value of lymphadenectomy (HR ═ 0.79) presents a new basis for clinical practice, possibly related to reduced tumor load or improved local control. Caution is warranted regarding selection bias, as surgical patients may have better baseline health status. Additionally, the improved prognosis for patients with primary intestinal involvement (HR ═ 0.89) may relate to the clearer presentation of symptoms and timeliness of diagnosis, although the potential impact of anatomical differences, such as blood supply and microenvironment, requires further investigation.

The nomogram model developed in this study outperforms the Ann Arbor staging system in both discrimination (C-index ═ 0.71) and calibration (C-index ═ 0.56), with DCA confirming significant clinical net benefits. This result is consistent with the trend of extensive application of nomogram in solid tumors [9, 25]. The advantages of the nomogram include the incorporation of treatment methods and social factors, providing a quantitative tool for individualized prognostic assessment. However, the model has limitations: first, the SEER database lacks key variables such as Eastern Cooperative Oncology Group (ECOG) performance status, lactate dehydrogenase (LDH) levels, and molecular typing (e.g., GCB/ABC subtype), which may affect the comprehensiveness of predictions. Second, the retrospective design cannot eliminate confounding factors, such as biases in treatment selection. Third, external validation of the model should include multi-center studies or diverse ethnic groups to enhance generalizability.

## Conclusion

In summary, through the analysis of large sample data, this study identified the independent effects of age, Ann Arbor stage, treatment methods, and social factors on the prognosis of gastrointestinal DLBCL, and successfully constructed the nomogram prediction model for this disease. The collected clinical variables include demographic characteristics, disease characteristics, and treatment information, with more stratified analysis used for marital status, Ann Arbor staging, race, and treatment methods. Multivariate Cox regression analysis suggested that age >60 years old (HR ═ 2.85), Ann Arbor stage (stage II: HR ═ 1.22; Phase III: HR ═ 1.31; Stage IV: HR ═ 1.85) and widowed status (HR ═ 1.40) are independent poor prognostic factors. Different treatment methods include chemotherapy (HR ═ 0.37), radiotherapy (HR ═ 0.84), surgery (HR ═ 0.86), and lymph node dissection (HR ═ 0.79), exhibited significant survival benefits. Intestinal primary (HR ═ 0.89), white race (HR ═ 0.78) and other races (HR ═ 0.65) were also associated with better prognosis. LASSO regression analysis selected age (β ═ 0.774, positively correlated with risk factors) and chemotherapy (β ═ −0.733, negatively correlated with risk factors) as the two most significant variables, which was consistent with the multivariate Cox regression analysis results. Through the analysis of the interaction between radiotherapy, chemotherapy, surgery, LNR, age, and other variables, which shown that the primary site and Ann Arbor staging are prone to interactive effects with other variables. The nomogram model in both training and validation cohorts exhibited excellent predictive performance with a C-index of 0.71, that is significantly better than traditional Ann Arbor staging system (C-index ═ 0.56). Moreover, stratified analysis of multivariate survival curves can effectively distinguish survival risks of patients. Therefore, this model providing support for identifying high-risk patients, adjusting treatment intensity, and optimizing follow-up strategies, and can quantitatively evaluate the survival probability of individual patients. Future researches should focus on improving models through prospective studies and integrating molecular biomarkers to promote the application of precision medicine in the field of gastrointestinal lymphoma.

## Supplemental data

Supplemental data are available at the following link: https://www.bjbms.org/ojs/index.php/bjbms/article/view/12697/3956.

## Data Availability

The original data is obtained from SEER database. All analysis data in this study are available from the corresponding or first authors on reasonable request.
